# Endogenous APOBEC3B Overexpression Constitutively Generates DNA Substitutions and Deletions in Myeloma Cells

**DOI:** 10.1038/s41598-019-43575-y

**Published:** 2019-05-09

**Authors:** Hiroyuki Yamazaki, Kotaro Shirakawa, Tadahiko Matsumoto, Shigeki Hirabayashi, Yasuhiro Murakawa, Masayuki Kobayashi, Anamaria Daniela Sarca, Yasuhiro Kazuma, Hiroyuki Matsui, Wataru Maruyama, Hirofumi Fukuda, Ryutaro Shirakawa, Keisuke Shindo, Masaki Ri, Shinsuke Iida, Akifumi Takaori-Kondo

**Affiliations:** 1 Department of Hematology and Oncology, Graduate School of Medicine, Kyoto University, Kyoto, 606-8507 Japan; 2 RIKEN-HMC Clinical Omics Unit, RIKEN Baton Zone Program, Kanagawa, 230-0045 Japan; 3 RIKEN Preventive Medicine and Diagnosis Innovation Program, Kanagawa, 230-0045 Japan; 4 Department of Molecular and Cellular Biology, Institute of Development, Aging and Cancer, Tohoku University, Sendai, 980-8575 Japan; 5 Department of Hematology and Oncology, Nagoya City University Graduate School of Medical Sciences, Nagoya, Japan

**Keywords:** Myeloma, Oncogenes

## Abstract

Apolipoprotein B mRNA-editing enzyme catalytic polypeptide-like (APOBEC) DNA cytosine deaminases have emerged as potential genomic mutators in various cancers. Multiple myeloma accumulates APOBEC signature mutations as it progresses; however, the mechanisms underlying APOBEC signature acquisition and its consequences remain elusive. In this study, we examined the significance and clinical impact of APOBEC3B (A3B) activity in multiple myeloma. Among APOBECs, only highly expressed A3B was associated with poor prognosis in myeloma patients, independent of other known poor prognostic factors. Quantitative PCR revealed that CD138-positive primary myeloma cells and myeloma cell lines exhibited remarkably high A3B expression levels. Interestingly, lentiviral A3B knockdown prevented the generation of deletion and loss-of-function mutations in exogenous DNA, whereas in control cells, these mutations accumulated with time. A3B knockdown also decreased the basal levels of γ-H2AX foci, suggesting that A3B promotes constitutive DNA double-strand breaks in myeloma cells. Importantly, among control shRNA-transduced cells, we observed the generation of clones that harboured diverse mutations in exogenous genes and several endogenous genes frequently mutated in myeloma, including *TP53*. Taken together, the results suggest that A3B constitutively mutates the tumour genome beyond the protection of the DNA repair system, which may lead to clonal evolution and genomic instability in myeloma.

## Introduction

Multiple myeloma (MM) is a plasma cell malignancy that harbours a wide variety of genetic alterations, including base substitutions, translocations, copy number variations and aneuploidy^1,2^. MM develops from monoclonal gammopathy of undetermined significance (MGUS) by accumulating genomic DNA mutations during physiological B cell maturation^3^. According to the classic karyotypic classification system, MM/MGUS is divided into two subtypes: hyperdiploid, which is characterized by multiple trisomies of odd-numbered chromosomes and a lack of recurrent immunoglobulin gene translocations, and non-hyperdiploid, which is characterized by chromosome translocations t(4;14), t(14;16), t(14;20), t(6;14) and t(11;14)^4^. Among these karyotypic changes, ectopic expression of cyclin D is relatively often detected, either directly through its juxtaposition to an immunoglobulin enhancer and copy number amplification or indirectly through unidentified mechanisms^4^. Most of the translocations are caused by errors in immunoglobulin heavy chain (IgH) class switch recombination and V(D)J recombination^3^, while the rest are caused by errors in somatic hypermutation during plasma cell development in germinal centers^5^. Thus, these translocations are referred to as primary translocations and are considered the primary steps of oncogenesis in normal plasma cells^6^. Based on its expression profiles, MM is divided into seven signature groups similar to its cytogenetic classifications: CD-1 and CD-2 (CCND1/CCND3), HY (hyperdiploid), LB (low bone disease), MS (MMSET), MF (c-MAF/MAF-B) and PR (proliferation). These signature groups are correlated with clinical prognosis: MS, MF and PR are correlated with high risk and CD-1, CD-2, HY, and LB are correlated with low risk^7^. DNA hypomethylation, deletion of chromosome 13, MYC dysregulation, and driver mutations of *RAS* and *BRAF* are considered signs of malignant progression^8^. Particular translocations of the *MUM1*/*IRF4* (6p25), *MAFB* (20q11), *IRTA2* (1q21) and *MYC* (8q24) loci, which rarely involve immunoglobulin genes, are correlated with poor clinical outcomes^6^. Furthermore, during MM progression and relapse, additional genetic abnormalities such as dysregulation of the NF-κB pathway, loss of chromosome 17p and/or abnormalities of TP53 develop and contribute to achieving independence from the bone marrow microenvironment^4,8^.

As with many other cancers, the presence of different subclones within MM tumours that are characterized by distinct genetic mutations independently contributes to MM progression^9^. High levels of intra-tumoural clonal heterogeneity and alterations in clonal dominance under therapeutic selective pressure have been described in patients with high-risk MM^10^. Hence, the molecular events underlying myeloma development and progression do no proceed in a linear fashion but rather through a Darwinian branching model^9–11^. Nevertheless, the causes of these events are largely unknown. Although activation-induced cytidine deaminase (AID) is considered to be responsible for early oncogenic processes, i.e., initiation of MM/MGUS, myeloma cells usually do not express AID^12^ except when interacting with dendritic cells^13^. Strikingly, whole-genome sequencing has revealed that MM contains apolipoprotein B mRNA-editing enzyme catalytic polypeptide-like (APOBEC) signature mutations^11,14–16^. Accumulation of APOBEC signature mutations increases significantly during tumour recurrence and extramedullary extension^11^ and is associated with poor prognosis^16,17^. Moreover, kataegis, which is defined by hypermutation in localized genomic regions and is supposedly generated by APOBECs^18^, has been found at *MYC*/*IGK* or *IGL* translocation breakpoints^16^, suggesting the co-occurrence of chromosomal translocations and APOBEC-associated mutations.

APOBEC3B (A3B) is an APOBEC cytidine deaminase that plays critical roles in immunity and is now highlighted as an intrinsic mutagen of genomic DNA that induces C-to-T and C-to-G substitutions, especially in breast cancer^19–22^. Among the seven APOBEC3 enzymes (APOBEC3A/B/C/DE/F/G/H; A3A–A3H), A3B is the only family member that is predominantly located in the nucleus throughout the cell cycle^23^. We previously reported that A3B induces C-to-T transitions in genomic DNA in human cell culture models^24^; therefore, we hypothesized that A3B might also induce DNA mutations in MM. In this study, we investigated the mutagenic activity of A3B in myeloma cells, and we here report how aberrantly expressed A3B induces DNA mutations and deletions and affects the survival of MM patients.

## Results

### A3B expression is aberrantly high in most malignant plasma cell samples from MM/MGUS patients and is associated with poor prognosis

**Figure 1:**
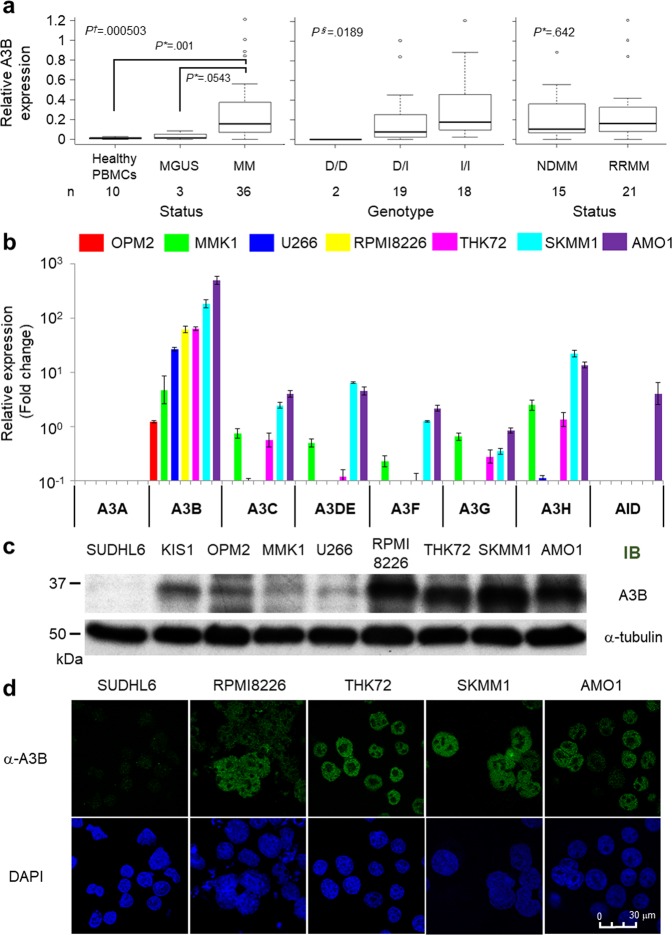
APOBEC family gene expression levels in MM/MGUS patients and myeloma cell lines. (**a**) Real-time PCR analysis of CD138-positive cells from MM/MGUS patient bone marrow. Statistical analysis of the correlation between A3B mRNA levels and diagnosis (healthy vs MGUS vs MM), genotype (D/D vs D/I vs I/I), or disease status (newly diagnosed MM [NDMM] vs relapse/refractory MM [RRMM]). The relative quantity of A3B mRNA was normalized by HPRT1 mRNA. *P* values were calculated using the Mann-Whitney U test (*), Kruskal-Wallis test (†) or Jonckheere-Terpstra test (§). (**b**) Real-time PCR of each of the APOBEC3 family genes (from A3A to A3H) and AID in seven myeloma cell lines (OPM2, MMK1, U266, RPMI8226, THK72, SKMM1 and AMO1). The target mRNA expression levels were normalized by HPRT1 mRNA levels. The target mRNA levels from PBMCs were used as a reference. (**c**) Immunoblot analysis of A3B in SUDHL6 cells (negative controls), KIS1 cells (positive controls) and the seven myeloma cell lines. α-Tubulin was evaluated as an internal control. (**d**) Fluorescence immunostaining using an anti-A3B antibody in SUDHL6, RPMI8226, THK72, SKMM1 and AMO1 cells. The images were obtained by confocal fluorescence microscopy (magnification, 630x).

First, we investigated the expression levels and genotypes of A3B in samples from MM/MGUS patients in our institutes. The patient characteristics are shown in Supplemental Table 1. MGUS patients accounted for 22.0% (n = 20), newly diagnosed multiple myeloma (NDMM) patients accounted for 45.1% (n = 41) and relapse/refractory MM (RRMM) patients accounted for 33% (n = 30) of a total of 91 patients. For 39 patients, we obtained the RNA of CD138+ myeloma cells from bone marrow samples to examine A3B expression. Because it was very difficult to obtain sufficient CD138-sorted plasma cells, PBMCs from healthy individuals were used as negative controls. Quantitative PCR analysis showed remarkably high expression levels of A3B in the majority of MM/MGUS patients (range, 0 to 1.214; median, 0.991; control vs MM/MGUS, *P* = 0.00397; control vs MM, *P* = 0.001; Fig. 1a, left panel). Regarding A3B genotypes, a deletion polymorphism that removes the entire A3B gene has been reported, and its frequency varies among major continental regions^25,26^. We examined the frequency of the A3B deletion allele in our patients and found that 44 were wild type (I/I, 48.4%; 95% CI, 37.7–59.1%), 40 were heterozygous (D/I, 44.0%; 95% CI, 33.6–54.8%), and 7 were homozygous for the deletion allele (D/D, 7.7%; 95% CI, 3.1–15.2%). There was no significant difference in allele frequency between MM/MGUS patients and Japanese healthy controls^26^ (Supplemental Table 2), although the A3B deletion allele tended to be more prevalent in MGUS (15.0%; 95% CI, 3.2–37.9%) than in MM (5.6%; 95% CI, 1.6–13.8%) patients (Supplemental Table 2). A3B expression levels were significantly correlated with A3B genotype (*P* = 0.0189; Fig. 1a, middle panel) and showed a weak positive correlation with diagnosis (MGUS vs MM, *P* = 0.0543; Fig. 1a, left panel) but were not correlated with disease status (NDMM vs RRMM, *P* = 0.642; Fig. 1a, right panel).

Next, to investigate the clinical impact of each APOBEC, we analysed a microarray dataset of 414 NDMM patients from Arkansas University^7^. Among the APOBEC genes whose probes were available in the platform of the study, A3B expression reached very high levels (Supplemental Fig. 1a), although A3B is generally not expressed in normal tissues^27^. It has been recently described that patients with an absolute APOBEC signature contribution at diagnosis in the fourth quartile have worse progression-free survival and overall survival (OS) than patients in the first to third quartiles^17^. Thus, we divided the cohort into two groups: a high-APOBEC group including patients whose expression of each APOBEC as determined by microarray data was over the fourth quartile limit and a low-APOBEC group including patients with APOBEC expression under that limit. Importantly, only A3B was correlated with a significantly worse OS (A3B high group: 3-year OS, 66.2%; 95% confidence interval [CI], 52.9–76.5% vs A3B low group: 3-year OS, 81.8%; 95% CI, 75.6–86.5%; *P* = 0.00133; Supplemental Fig. 1b). Because A3B expression is correlated with known risk factors^16^, we also assessed OS after risk group stratification based on genetic signatures^7^ to exclude potential confounding biases. Consistent with a previous report^16^, A3B expression differed significantly between the molecular subgroups (Supplemental Fig. 1c, *P* = 1.17 × 10^−14^). Univariate analysis showed that A3B expression levels still had a significant prognostic impact in the high-risk group (Supplemental Fig. 1d, right panel, *P* = 0.00357) as well as over long-term observation periods in the low-risk group (Supplemental Fig. 1d, left panel; *P* = 0.143, logrank test and *P* = 0.00357, 45-month landmark analysis). Univariate analysis also detected high A3C, high A3DE and low A3F as significant risk factors when the median was used as the threshold (Supplemental Table 3). However, only known risk and high A3B remained independent risk factors for OS after multivariate analysis following a Cox regression model (known high-risk group: hazard ratio [HR], 2.342; 95% CI, 1.503–3.649; *P* = 0.0001693 and high A3B: HR, 2.057; 95% CI, 1.294–3.270; *P* = 0.0002305; Supplemental Table 3).

### A3B is highly upregulated and localized at the nucleoplasm in MM cell lines

We next investigated the expression profiles of AID and APOBEC3 family members in seven myeloma cell lines: OPM2, MMK1, U266, THK72, RPMI8226, SKMM1 and AMO1. Consistent with the results from primary myeloma cells, these cell lines also expressed prominently high levels of A3B, as determined by quantitative PCR analysis (quantity relative to that in PBMCs: range, 1.22 to 489.4; median, 62.47; Fig. 1b). Cap analysis of gene expression (CAGE) in these seven myeloma cell lines showed that the activity of both A3B promoters was aberrantly upregulated compared to that in CD19+ cells from healthy individuals (Supplemental Fig. 2). We next determined protein expression levels using an anti-A3B antibody that we generated by immunizing rabbits with a C-terminal A3B peptide^28^. Because of the high homology of the C-termini, this anti-A3B antibody also detects artificially overexpressed A3A and A3G (Supplemental Fig. 3a,b). However, at endogenous expression levels, the antibody is more specific for A3B than for A3G (Supplemental Fig. 3c). We could also distinguish among these three proteins based on their sizes in immunoblot analyses (A3A, 25 kDa; A3B, 35 kDa; A3G, 40 kDa) and by their subcellular localization in immunofluorescence assays (A3A, cytoplasm and nucleus; A3B, nucleus; A3G, cytoplasm) (Supplemental Fig. 3a–c). We demonstrated that there was aberrant expression of A3B at the protein level in each MM cell line by immunoblotting (Fig. 1c) and confirmed A3B expression in the nuclei, but not in the nucleoli or in the cytoplasm, by using immunofluorescence assays (Fig. 1d); these findings were consistent with previous evidence^23^.

### Endogenous A3B overexpression contributes to loss of function of lentivirally introduced genomic DNA and to constitutive DNA double-strand breaks in myeloma cells

**Figure 2:**
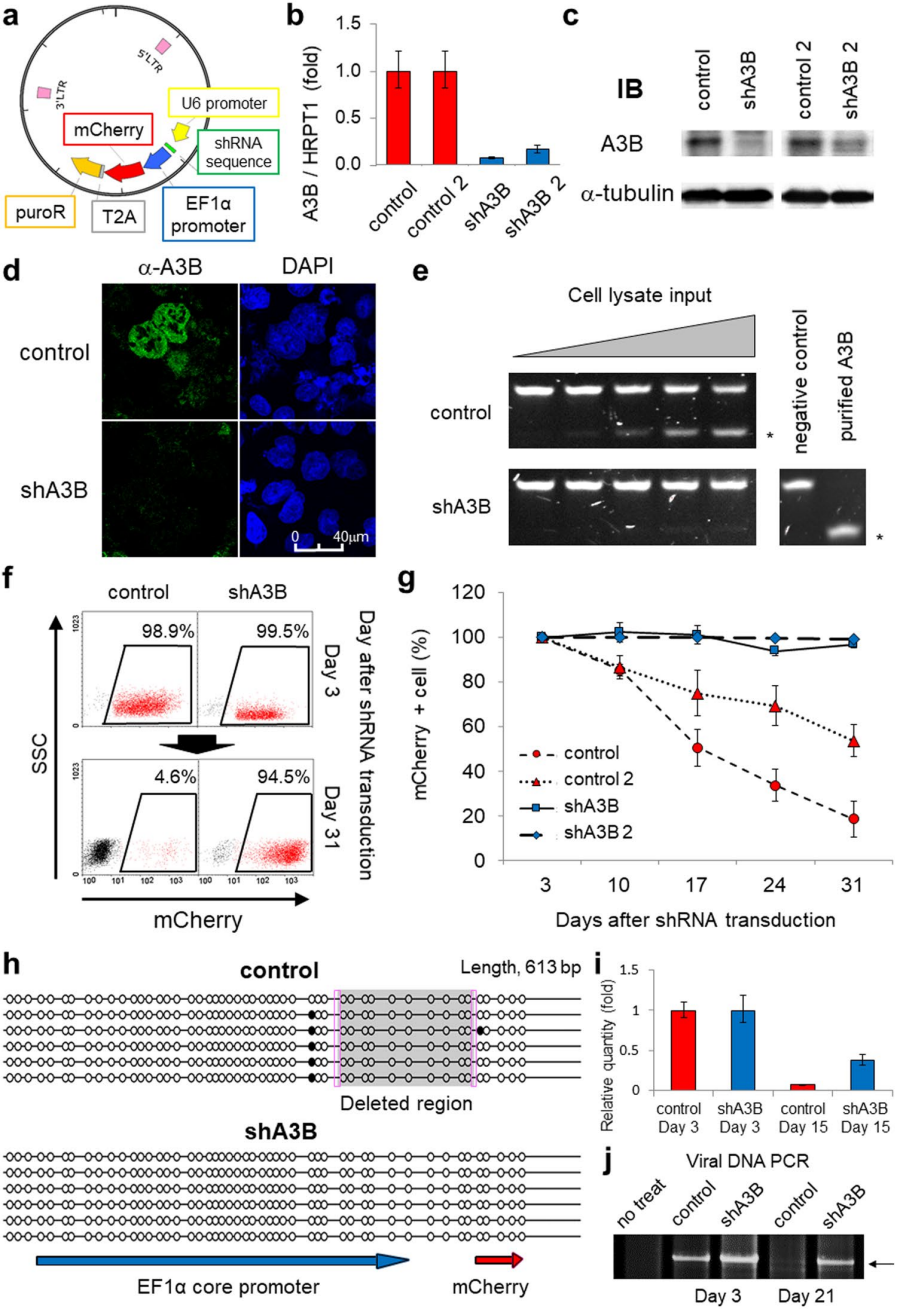
APOBEC3B mediates the loss of function of exogenous genes in myeloma cells. (**a**) Schema of the shRNA lentiviral vector construct. The produced lentivirus transduces shRNA together with mCherry and the puromycin resistance gene (puroR). (**b**,**c**) Real-time PCR (**b**) and immunoblotting (**c**) results of A3B levels in RPMI8226 cells, which were transduced with lentiviral shRNA against A3B (two constructs: shA3B and shA3B-2) or control shRNA (two constructs: control and control-2). HPRT1 or α-tubulin was evaluated as an internal control. (**d**) Immunofluorescence analysis using an anti-A3B antibody of RPMI8226 cells transduced with either shRNA against A3B or control lentivirus. The images were obtained by confocal fluorescence microscopy (magnification, 630x). (**e**) *In vitro* assay for cytidine deaminase activity in RPMI8226 cells transduced with either shRNA against A3B or control lentivirus. The asterisks indicates the cleaved DNA products. (**f**) Flow cytometry of RPMI8226 cells at 3 and 31 days after transduction with each mCherry-shRNA lentivirus. The numbers in boxes indicate the proportions of mCherry-positive cells among the live cells for each condition. (**g**) Time-dependent changes in the proportions of mCherry-positive cells among live cells transduced with each shRNA lentivirus as determined by flow cytometry. (**h**) Representative figure of the EF1α promoter methylation assay. The black circles represent methylated CpG. The shaded box indicates the deleted region. The sense strands of the EF1α promoter and a portion of the mCherry gene region are shown for reference. The pink boxes indicate microhomology at the DNA double-strand breakpoint. (**i**) Real-time PCR of genomic mCherry. RPMI8226 genomic DNA expression at 3 and 15 days after transduction with each shRNA lentivirus was examined. *APOB* was evaluated as an internal control. The value at day 3 was used as a reference. (**j**) Conventional PCR of the full-length viral vector DNA. RPMI8226 genomic DNA expression at 3 and 21 days after transduction with each shRNA lentivirus was examined. The arrow indicates the amplicon size between the two LTR regions of the lentiviral vector (5200 bp).

To assess the mutagenic activity of endogenous A3B in MM cells, we transduced RPMI8226 cells with lentiviral shRNA against A3B (shA3B) and with an EF1α-driven mCherry fluorescent marker (Fig. 2a). We confirmed A3B depletion at the mRNA and protein levels (Fig. 2b–d) and confirmed that there was little cytidine deaminase activity in shA3B-transduced cells (Fig. 2e). Notably, lentiviral shA3B transduction also efficiently decreased endogenous A3B expression in other MM cell lines, such as THK72, SKMM1 and AMO1, which are susceptible to lentivirus infection (Supplemental Fig. 3d,e). Interestingly, control shRNA-transduced cells lost mCherry fluorescence over a span of three weeks, whereas shA3B-transduced cells stably maintained it in the RPMI8226 cell line (Fig. 2f,g) as well as the THK72 and AMO1 cell lines (Supplemental Fig. 4a–d). To investigate this fluorescence loss, we determined the DNA methylation status of the EF1α promoter by bisulfite sequencing. DNA methylation was absent in both control shRNA- and shA3B-transduced cells, indicating that DNA methylation-mediated silencing did not cause the loss of fluorescence. Instead, we detected a large deletion accompanied by 6 bp microhomology (i.e., CGCCGT) at the junction point in the EF1α promoter in control shRNA-transduced cells (Fig. 2h). Using real-time PCR to quantify the copy number of the transduced mCherry genes, we detected a faster decrease in control shRNA-transduced cells than in shA3B-transduced cells (Fig. 2i). In addition, amplification of the full-length shRNA construct failed at day 21 in control shRNA-transduced cells (Fig. 2j). These data support the premise that the loss of fluorescence was caused by deletion mutations and/or ablation of the intact transduced gene.

**Figure 3:**
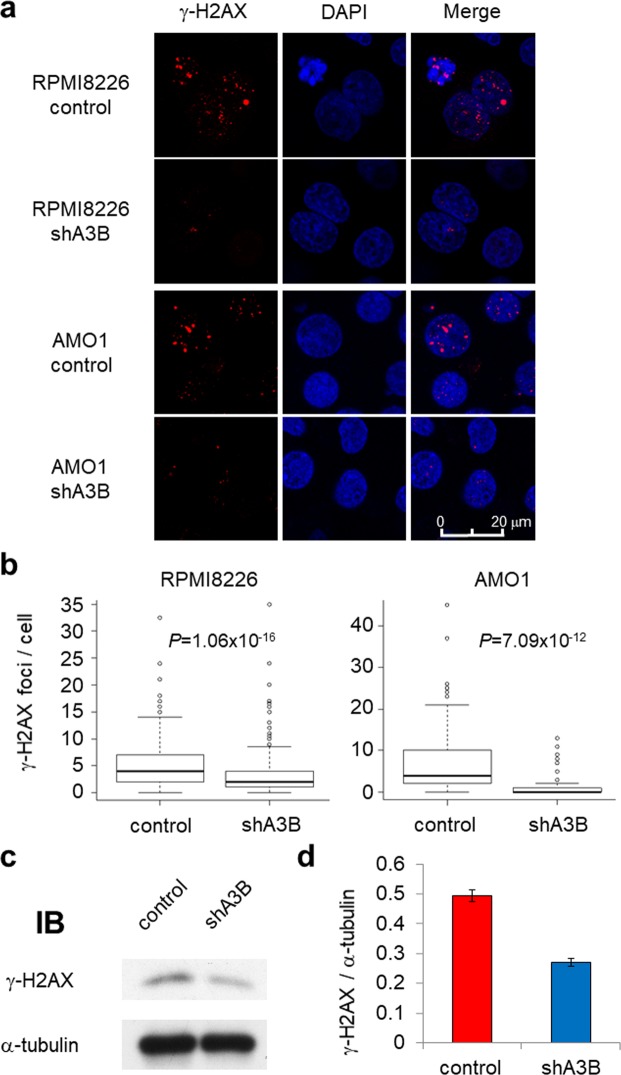
APOBEC3B promotes DNA double-strand breaks in myeloma cells. (**a**) Fluorescence immunostaining of γ-H2AX in RPMI8226 and AMO1 cells transduced with either control or A3B shRNA lentivirus. The images were obtained by confocal fluorescence microscopy (magnification, 630x). (**b**) Statistical analysis of the number of γ-H2AX foci per cell in RPMI8226 and AMO1 cells based on the images in (**a**). Approximately 200 cells were evaluated for each sample. All *P* values were calculated using Student’s t test. (**c**) Immunoblot analysis of γ-H2AX in RPMI8226 cells transduced with either control or A3B shRNA lentivirus. (**d**) Bar graph of the γ-H2AX band intensities from (c) normalized by the α-tubulin band intensity.

Myeloma cells have been reported to exhibit constitutive DNA double-strand breaks (DSBs)^29^; therefore, we next investigated whether A3B knockdown changes the status of γ-H2AX, a DSB marker. shA3B transduction significantly reduced γ-H2AX protein levels, which was confirmed by fluorescence immunostaining in RPMI8226 and AMO1 cells (Fig. 3a,b) and immunoblot analysis in RPMI8226 cells (Fig. 3c,d). These results suggest that A3B contributes to constitutive DSBs leading to gene alterations in myeloma cells.

### A3B constantly generates C>T|G>A transitions, which are then processed by the DNA repair system

**Figure 4:**
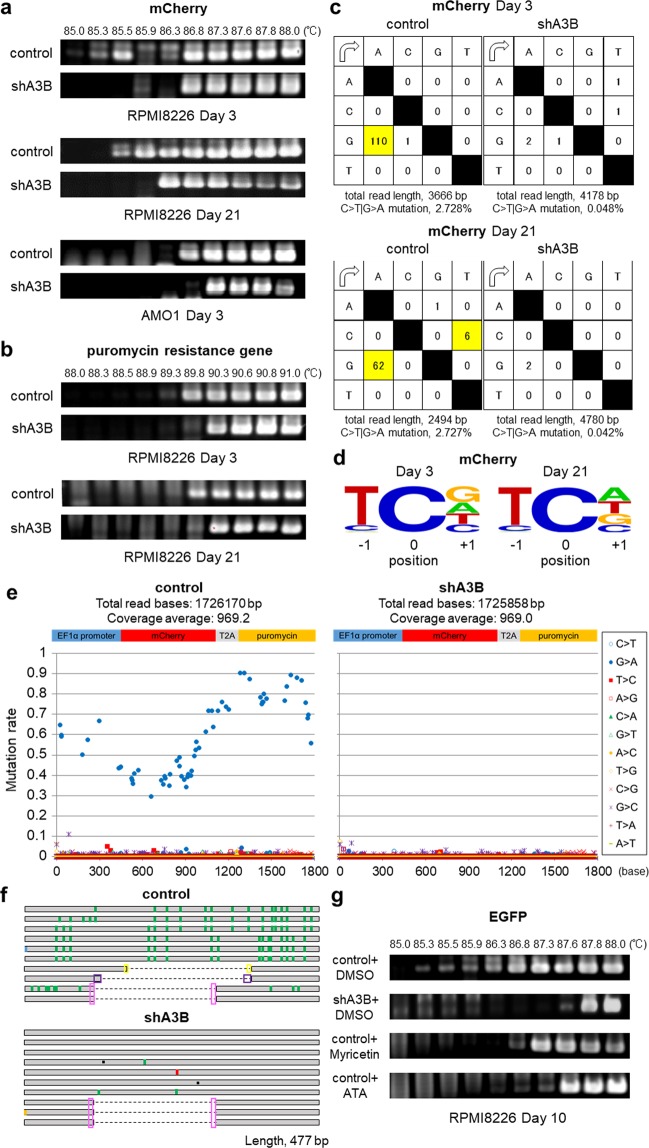
APOBEC3B induces C>T|G>A mutations in lentivirally introduced genomic DNA in myeloma cells. (**a**,**b**) 3D-PCR analysis of mCherry genes (**a**) and puromycin resistance genes (puroR) (**b**) derived from RPMI8226 genomic DNA at 3 or 21 days post transduction with each shRNA lentivirus. 3D-PCR analysis of mCherry genes derived from AMO1 genomic DNA at 3 days post transduction with each shRNA lentivirus is also indicated in (**a**). (**c**) Mutation matrices of hyperedited mCherry sequences derived from RPMI8226 genomic DNA at 3 (upper panel) and 21 (lower panel) days post transduction with each shRNA lentivirus. The sequence data were obtained by performing TA cloning and Sanger sequencing. The first column indicates the bases before mutation, and the first line indicates the bases after mutation. The highlighted boxes indicate significant C-to-T or G-to-A substitutions. The sense strand of the mCherry sequence was used as a reference. (**d**) Sequence logo created with WebLogo indicating the frequencies of nucleotides adjacent to C-to-T mutation sites. (**e**) Deep sequencing analysis of the 3D-PCR products of mCherry-T2A-puroR genes derived from RPMI8226 genomic DNA at 21 days post transduction with each shRNA lentivirus. The Y axis indicates the proportion of each substitution in the total coverage, and the X axis indicates its location in the amplified gene from the EF1α promoter to the puroR gene. (**f**) Schema of Sanger sequencing results of each of the 10 clones of mCherry 3D-PCR products at 3 days post transduction with each shRNA lentivirus. G-to-A substitutions are indicated in green, C-to-T substitutions in red, G-to-C substitutions in light blue, and A-to-T substitutions in orange. The black squares indicate single-base deletions, whereas the dotted lines represent large deletions. The pink, purple or yellow boxes indicate microhomology at the DNA double-strand breakpoint. (**g**) 3D-PCR of *EGFP* derived from RPMI8226 genomic DNA at 10 days post transduction with control shRNA lentivirus under continuous incubation with the APOBEC3 inhibitors myricetin (5 μM) and aurintricarboxylic acid (ATA, 1 μM). We used lentiviral shRNA against APOBEC3B as a positive control.

We further tested the mutagenic activity of A3B by differential DNA denaturation PCR (3D-PCR), which efficiently amplifies DNA containing C>T|G>A transitions^30^. The mCherry sequences were amplified at lower denaturation temperatures in control shRNA-transduced than in shA3B-transduced RPMI8226 and AMO1 cells, both at Day 3 and at Day 21 after transduction (Fig. 4a). 3D-PCR of the puromycin resistance gene (puroR) also produced similar results (Fig. 4b). Using TA cloning and Sanger sequencing, we confirmed prominent G-to-A mutations in the mCherry gene (Fig. 4c and Supplemental Table 4) that are typical of the APOBEC signature (Fig. 4d). Intriguingly, we detected only G-to-A mutations, indicating that C-to-U deamination preferentially occurs in the antisense strand of the gene (Fig. 4c and Supplemental Table 4). Deep sequencing of the 3D-PCR products of the mCherry-T2A-puroR gene demonstrated that only G-to-A transitions accumulated throughout the gene and that the frequency of mutations increased towards the 3′ region of the gene (Fig. 4e and Supplemental Fig. 5a). Of note, Sanger sequencing of the 3D-PCR products showed that the occurrence of the C>T|G>A transition was similar between Day 3 and Day 21 (Fig. 4c). In contrast, deep sequencing of conventional PCR products of the mCherry gene failed to detect substitutions (Supplemental Fig. 5b). These findings demonstrate that mutations amplified by 3D-PCR occur in the presence (and occur less in the absence) of A3B in at least some fraction of cells or that DNA repair pathways might repair A3B-mediated DNA lesions. In fact, the 3D-PCR products of both control and shA3B-transduced cells contained large deletions that had at least 3-base microhomology at the junction points, suggesting that these mutations are at least in partly repaired by the microhomology-mediated end-joining (MMEJ) machinery^31^ (Fig. 4f and Supplemental Table 4). The results of bisulfite sequencing of the EF1α promoter after conventional PCR also supported the involvement of MMEJ repair (Fig. 2h). Additionally, the combination of substitutions and deletions varied among the clones obtained from control cells, resulting in different amino acid sequences (Fig. 4f, Supplemental Tables 4 and 5). Abolition of the mCherry chromophore was predicted in clones that contained approximately hundreds of base deletions, suggesting that loss of mCherry fluorescence was at least partly caused by these deletion mutations (Supplemental Table 5).

We confirmed gene amplification at lower denaturation temperatures by 3D-PCR after shA3B transduction in two other A3B-expressing myeloma cell lines, including AMO1 (Fig. 4a, lower panel). Importantly, aurintricarboxylic acid and myricetin, which are known APOBEC3 deaminase inhibitors^32^, also allowed for 3D-PCR amplification of the mCherry gene at lower denaturation temperatures in myeloma cells (Fig. 4g), suggesting that this cell-based model could be used for screening APOBEC3B inhibitors.

**Figure 5:**
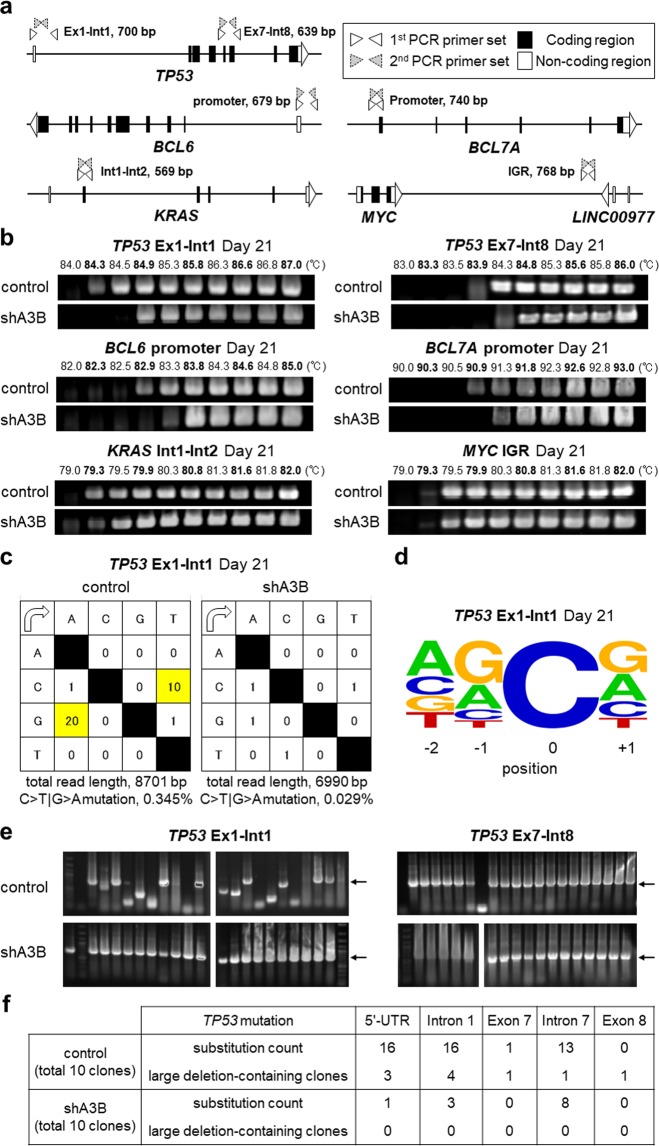
APOBEC3B exerts its mutagenic activity on endogenous genes. (**a**) Primer settings for 3D-PCR of the endogenous genes of interest. The white boxes indicate 5′- or 3′-UTR sequences, and the black boxes indicate protein-coding sequences. The white and grey triangles represent the first and second PCR primer sets, respectively. (**b**) 3D-PCR of particular loci in genomic DNA obtained from RPMI8226 cells at 21 days post transduction with control or shA3B lentivirus. The loci include *TP53* from exon 1 (Ex1) to intron 1 (Int1) and from exon 7 (Ex7) to intron 8 (Int8), the *BCL6* promoter, the *BCL7A* promoter, *KRAS* from intron 1 (Int1) to intron 2 (Int2), and the *MYC* intergenic region (IGR). (**c**) Mutation matrices of hyperedited *TP53* sequences from Ex1 to Int1 obtained from RPMI8226 genomic DNA at 21 days post transduction with each shRNA lentivirus. The sequence data were obtained by performing TA cloning and Sanger sequencing. The first column indicates the bases before mutation, and the first line indicates the bases after mutation. The sense strand of *TP53* was used as a reference. C-to-T and G-to-A substitutions are highlighted. (**d**) Sequence logo created with WebLogo indicating the frequencies of nucleotides adjacent to C-to-T mutation sites in *TP53* Ex1-Int1. (**e**) Representative colony PCR results for *TP53* from Ex1 to Int1 (left) and from Ex7 to Int8 (right) after TA cloning. Clones derived from control- or shA3B- transduced cells were selected from the PCR products obtained at the lowest denaturation temperatures in (**b**). The arrows indicate the intact sizes of the nested PCR products for *TP53* (700 bp and 639 bp). (**f**) Summary of Sanger sequencing for the 3D-PCR products of *TP53* Ex1-Int1 and Ex7-Int8.

Lastly, we examined the state of several endogenous genes that are known to be frequently mutated (e.g., *TP53* and *KRAS*) or in which breakpoints are observed in translocations frequently occurring in myeloma cells (e.g., in the *MYC* intergenic region)^8^ (Fig. 5a). APOBEC signature mutations are known to be associated with DNA breakpoints^20^. Promoter regions of *BCL6* and *BCL7A* were also selected because cooperation between A3B and transcription factors has been reported^33^. Intriguingly, we detected significant 3D-PCR amplification of genes such as *TP53* and the *BCL6* and *BCL7A* promoters but little difference in *KRAS* (Fig. 5b). These data are consistent with previous reports showing that *TP53*-inactivating mutations are commonly found throughout the *TP53* gene and that *KRAS*-activating mutations are limited to hot spots (G12/G13/Q61/A146)^34^. We did not detect significant amplification in MYC intergenic regions, possibly because the MYC breakpoint is found in the megabase-wide intergenic region of *MYC*^35^, while our primer sets for MYC 3D-PCR analysis only covered 700 and 679 base pairs (Fig. 5a). Sanger sequencing of the mutated *TP53* genes confirmed C>T|G>A transitions (Fig. 5c,d) and deletions (Fig. 5e), as summarized in Fig. 5f. We confirmed that these G>A|C>T transitions in *TP53* in the 3D-PCR product were reproducible in other cell lines, such as AMO1 and THK72 (Supplemental Fig. 6a–f). These results indicate that A3B mutates endogenous genes in genomic DNA as well as exogenous genes in transduced DNA, in our case the mCherry-T2A-puroR gene. These data suggest that most DNA substitutions induced by overexpressed A3B will be repaired by DNA repair pathways and that some clones will survive in the long term under selective pressure in myeloma cells.

## Discussion

The present study demonstrates that endogenous A3B overexpression constitutively produces various combinations of C>T|G>A mutations and promotes DSB-related gene alterations in myeloma cells. Recent studies have demonstrated that there are clinical consequences of APOBEC activity in malignant tumours^36^, especially in breast cancer^19,21^. Previous genome-wide analyses of MM/MGUS samples have also shown that mutation patterns associated with APOBEC activity correlate with disease progression and clinical outcomes^11,16,17^, whereas those associated with AID activity contribute to disease initiation^37^. In ER-positive breast cancer, high A3B expression has been reported to correlate with treatment resistance, metastasis and poor prognosis^22,38^, and there have been similar reports for lung cancer^39^ and ovarian cancer^40^. Our analyses also show that aberrant A3B expression could have an impact on prognosis in MM patients, as A3B may contribute to disease progression and drug resistance. The present study is the first to specifically investigate the activity of endogenous A3B and to demonstrate its on-going mutagenic effects in myeloma cells. Importantly, A3B knockdown profoundly decreased the cytidine deaminase activity of whole-cell lysates from myeloma cells, suggesting that among APOBECs, A3B plays a major role in cytidine deamination-related mutagenesis in myeloma cells (Fig. 2e). On the other hand, breast cancer also shows APOBEC signature mutations in the absence of A3B, intimating the involvement of other APOBEC proteins, such as A3A^41^ and A3H haplotype I^42^. A recent study showed that A3H haplotype I is associated with APOBEC signature mutations in lung and breast cancer^42^. We also found A3H to be relatively highly expressed in MM cell lines (Fig. 1b and Supplemental Fig. 2), suggesting the possible involvement of A3H-induced mutations in MM. Further studies are needed to confirm whether the A3H haplotype contributes to MM oncogenesis.

The precise mechanism of APOBEC-induced mutagenesis of genomic DNA is under intense investigation. Because APOBECs preferentially target single-stranded DNA (ssDNA), three situations have been proposed: first, that APOBECs target the lagging DNA strand under replication stress^43–45^; second, that they target ssDNA during the resection phase of homology-mediated repair after DSBs^20,36,43^; and third, that they target the non-transcribed strand during transcription^46–48^. Interestingly, in the present study, endogenous A3B deaminated cytosines mainly in the antisense strand (Fig. 4c,e and Supplementary Fig. 5a), suggesting that, instead, the transcribed strand might be the major A3B target. This discrepancy could have arisen due to differences in methodology; for example, 3D-PCR selectively amplifies minor clones of mutated DNA, whereas next-generation sequencing detects DNA mutations established through selection pressure or the DNA repair response.

Our data also suggested that lentivirally transduced genes lose their function due to deletions of hundreds of bases with microhomology at the junction points in A3B-overexpressing myeloma cells. It is plausible that multiplex attacks by A3B on ssDNA could cause DSBs, and subsequent MMEJ repair would produce a variety of clones harbouring deletions of various lengths. Whole-genome/exome sequencing with a coverage of tens to hundreds is usually able to detect only clones that account for more than a single-digit percentage of the total. Therefore, A3B-associated minor clones would be buried in sequencing errors and neglected. Since MMEJ repair of DSBs is undertaken at microhomology sequences near the breakpoint, clones with different DSB locations could be transformed into clones with the same deleted region. In fact, in our study, a number of clones with different combinations of mutations also had the same deleted region, as was revealed by TA cloning and Sanger sequencing (Figs 2h, 4f). However, most of the major mapping programs used for next generation sequencing, e.g., BWA and BWA-MEM^49,50^, do not cover deletions of hundreds of bases such as the large deletions in mCherry (195 bp at minimum) detected in our study. Paired-end read analysis can detect structural variance, including large deletions of thousands of bases; nevertheless, sequenced reads that contain approximately hundreds of base deletions are unlikely to be mapped and will generally be discarded. Methods to obtain comprehensive snapshots of the production of minor clones *in vivo* still need to be validated.

Regarding mutation of endogenous genes, we observed enrichment of C-to-T substitutions and deletions in intronic regions and non-coding exons in the endogenous *TP53* gene, consistent with previous reports^8^. Chapman *et al*. demonstrated that the mutation frequency in coding regions was lower than that observed in UTR/promoter, intronic and intergenic regions in MM samples^8^. DNA repair responses, including transcription-coupled repair, might preferentially correct mutations in coding regions^51^, or one or more unknown mechanisms might preferentially protect these regions from A3B attacks. Indeed, analysis of large whole-genome and exome datasets from bladder, cervical, breast, lung and head and neck cancers has indicated only a weak positive correlation between A3B expression and APOBEC-induced mutagenesis^36^. APOBEC-mediated mutations may develop as a result of the interplay between deaminase activity and the DNA damage response^52^. A limitation to using 3D-PCR for endogenous genes is the considerable PCR error rate. Suspene *et al*. stated that the 3D-PCR error rate is 0.02% owing to constant selection for AT DNA and PCR-mediated recombination^53^. In our study, the transition rate was 0.029% in the shA3B-derived genome, whereas in the control cells, the rate was elevenfold higher (0.345%, Fig. 5c). Accordingly, sequencing of the 3D-PCR products detected only background mutations in shA3B samples, but significantly more mutations were detected in control samples. Still, the mutational signature present in *TP53* appeared to be a complex cytosine mutation distribution rather than the typical A3B pattern (Fig. 5d). Previous studies on overexpressed A3B also showed similar results^24,54^, and the authors discussed the possibility that the intrinsic preference of A3B was skewed by downstream repair pathways or other mutation-generating processes^54^. Our data showing that mutational signatures were different between exogenous and endogenous genes despite the use of the same DNA inputs may support their hypothesis. Regarding the substitution rate, C>T|G>A transitions were much more frequent in the exogenous genes (2.728% in mCherry-T2A-puroR, Fig. 4c) than in the endogenous genes (0.345% in *TP53*, Fig. 5c) of control samples. One of the reasons behind these different rates might be that lentivirally transduced genes are processed by the DNA repair system differently than endogenous genomic genes. Further studies are needed to clarify this issue.

In conclusion, aberrantly highly expressed endogenous A3B can contribute to genomic instability and therefore to linear and branched clonal evolution in the context of long-term disease progression. Although the clinical impact of A3B-related oncogenesis in myeloma remains to be further evaluated, inhibiting A3B activity could protect against disease deterioration and could be a new therapeutic option.

## Materials and Methods

### Clinical samples

All investigations have been conducted in accordance with ethical standards and have been approved by the Institutional Review Boards of Kyoto University and Nagoya City University, respectively. Written informed consent for the banking and subsequent research using their specimens, including genomic studies, was obtained from each patient. Bone marrow mononuclear cells were first obtained after centrifugation of bone marrow aspirates using the Lympholyte®-H (CEDARLANE) density gradient separation medium, and then myeloma cells were further isolated using the MACS CD138 positive cell isolation kit (Miltenyi Biotec). To assess the A3B polymorphism, the intact (I) and deletion (D) alleles were genotyped as previously described^55^.

### Cell lines and cell culture

Six human myeloma cell lines, OPM2, U266, RPMI8226, THK72^56^, SKMM1 and AMO1, and two B-cell lymphoma cell lines, SUDHL6 and KIS1^28^, were maintained in RPMI1640 (Nacalai) containing 10% FBS and 1% PSG (Invitrogen). The MMK1 myeloma cell line was established in our laboratory by culturing primary myeloma cells from the pleural effusion of a refractory myeloma male patient.

### Quantitative RT-PCR

Total RNA was extracted from clinical samples and cell lines using the High Pure RNA isolation kit (Roche). Complementary DNA was synthesized using the PrimeScriptR II 1^st^ strand cDNA Synthesis Kit (Takara) by random primer and oligo dT primer mixture. Real-time PCR was performed using the Thunderbird SYBR qPCR Mix (ToYoBo). Target gene expression levels were normalized by endogenous expression levels of HPRT1. To assess the copy number of introduced mCherry genes in lentivirally transduced cells, genomic DNA was extracted using the QuickGene DNA whole blood kit S (KURABO) and was evaluated and normalized by the endogenous APOB allele. All primers for real-time PCR are listed in Supplemental Table 6.

### CAGE analysis

Promoter-level gene expression analysis was performed using CAGE^57^. Total RNA from each cell line was isolated using the RNeasy Mini Kit (Qiagen) and assessed with the Agilent RNA6000 Nano Kit (Agilent Technologies). 5 µg of total RNA (RIN > 7, A260/280 and 260/230 ratios > 1.7) was used for CAGE library preparation. CAGE libraries were made based on no-amplification non-tagging CAGE libraries for Illumina next-generation sequencers (nAnT-iCAGE) as previously described^57^. After Illumina sequencing, we used the MOIRAI pipeline^58^ to remove ribosomal RNA (rRNA) sequences and sequences with base ‘N’, and to align the reads. In the pipeline, rRNAdust (http://fantom.gsc.riken.jp/5/suppl/rRNAdust/) was used to remove rRNA with rRNA sequences (U13369.1) and the parameters of −e 2 and −t 8. Mapping to the hg19 reference genome was done using bwa -aln (version 0.5.9)^49^ with the parameters of −n 0.02, −o 1, −e −1, −i 5, −d 10, −l 32, −k 2, −m 2000000, −t 8, −M 3, −O 11, −E 4, −R 30, −q 0. Using samtools version 0.1.8^59^, the resultant SAM files were converted to BAM files and low-quality nucleotides (MAPQ < 10) were filtered out. 5′ ends of reads were counted on FANTOM5-defined CAGE peaks^60^ based on the annotation file retrieved on April 25th, 2017 from the link below. Tags per million (TPM) normalized expression values were used. (http://fantom.gsc.riken.jp/5/datafiles/latest/extra/CAGE_peaks/hg19.cage_peak_phase1and2combined_ann.txt.gz.)

### Immunoblot analysis

Whole cell lysates were subjected to immunoblot analysis using a purified rabbit anti-A3B polyclonal antibody, a mouse anti-phospho-histone H2A.X (Ser139) antibody (Millipore, clone JBW301) or a mouse anti-α-tubulin monoclonal antibody (AA13, Funakoshi). The anti-A3B antibody was generated from a rabbit immunized with the C-terminal peptide EEHSQALSGRLRAILQNQGN by Sigma Aldrich as previously described^28^.

### Knockdown experiments

We constructed pSicoR-mCherry/EGFP lentiviral vectors^61^ expressing short-hairpin RNA (shRNA) against A3B by inserting synthetic double-stranded oligonucleotides (TRCN0000140546^19^, sense oligo, 5′-TGCAAAGCAATGTGCTCCTGATCTCGAGATCAGGAGCACATTGCTTTGCTTTTTTC-3′, and antisense oligo, 5′-TCGAGAAAAAAGCAAAGCAATGTGCTCCTGATCTCGAGATCAGGAGCACATTGCTTTGCA-3′; TRCN0000139463, sense oligo, 5′-TCCTGATGGATCCAGACACATTCTCGAGAATGTGTCTGGATCCATCAGGTTTTTTC-3′, and antisense oligo, 5′-TCGAGAAAAAACCTGATGGATCCAGACACATTCTCGAGAATGTGTCTGGATCCATCAGGA-3′) into the cloning site. For non-target shRNA, we used two constructs that were cloned as scrambled sequences (control^62^, sense oligo, 5′-TGTCAAGTCTCACTTGCGTCTTCAAGAGAGACGCAAGTGAGACTTGACTTTTTTC-3′, antisense oligo, 5′-TCGAGAAAAAAGTCAAGTCTCACTTGCGTCTCTCTTGAAGACGCAAGTGAGACTTGACA-3′; control-2^63^, sense oligo, 5′-TATCTCGCTTGGGCGAGAGTAAGCTCGAGCTTACTCTCGCCCAAGCGAGATTTTTTTC-3′, antisense oligo, 5′-TCGAGAAAAAAATCTCGCTTGGGCGAGAGTAAGCTCGAGCTTACTCTCGCCCAAGCGAGATA). The lentivirus was produced by co-transfection of Trans-Lentiviral packaging plasmid mix (GE Dharmacon) and pSicoR-mCherry/EGFP into Lenti-X cells. Physical particle titers were measured using the RETRO-TEK HIV-1 p24 Antigen ELISA kit (ZeptoMetrix) and matched titers of each lentiviral vector (control vs shA3B) were used.

### A3B catalytic domain biochemistry

The DNA deamination assay was performed as previously described^64^. Cell lysates were mixed with a 6-FAM labeled 43-long oligonucleotide containing a TTCC deamination site for 30 min at 37 °C before adding uracil DNA glycosylase and NaOH to create and break an abasic site. The samples were separated on 15% acrylamide-urea gels and analyzed with the Gel Doc EZ system.

### 3D-PCR and Deep sequencing

For DNA editing assays, genomic DNA was extracted as described above from myeloma cells at 3 and 21 days after transduction by pSicoR-mCherry shRNA lentivirus. We performed 3D-PCR^30^ by amplifying genes using the KOD FX Neo (ToYoBo) DNA polymerase as described before^24^. All primer sets and conditions are listed in Supplemental Table 6. For Sanger sequencing, amplicons derived at the lowest temperature were incubated with A-attachment Mix (ToYoBo) and were subsequently cloned into the T-vector pMD20 (TaKaRa). Each extracted plasmid from DH5α cells (ToYoBo) transformed with the cloned vector was sequenced using the 3130xl Genetic Analyzer (Applied Biosystems). For deep sequencing analysis, amplicons (5 ng) derived at the lowest temperature were sheared and sequenced on an Illumina NextSeq instrument to obtain 75 nucleotides paired end reads. After Illumina sequencing, low-quality nucleotides (MAPQ < 10) were discarded and then Illumina sequencing adapters and primers sequences were removed from the reads. Finally, the reads were aligned using the BWA-MEM algorithm with default settings (version 0.7.12)^50^.

### Immunofluorescence assays

Myeloma cells were air-dried and fixed in 4% paraformaldehyde in phosphate-buffered saline (PBS) for 20 minutes on glass slides using Shandon cytospin 2 (THERMO FISHER SCIENTIFIC). Fixed cells were permeabilized, reduced and denatured for 30 minutes in PBS buffer containing 0.5% SDS, 5% β-mercaptoethanol and 10% FBS. Then, cells were washed three times with PBS containing 4% FBS and 0.1% Triton X-100 (PFT buffer)^65^, and incubated with a purified rabbit anti-A3B antibody for 1 hour. Subsequently, cells were incubated with a goat anti-rabbit IgG (H + L)-Alexa Fluor® 488 preadsorbed antibody (Abcam, ab150081) for 30 min in the dark. γ-H2AX foci analysis was performed as previously described^29^ using a mouse anti-phospho-histone H2A.X (Ser139) antibody (Millipore, clone JBW301) as primary antibody and a goat anti-mouse IgG (H + L)-Alexa Flour® 594 preadsorbed antibody (Abcam, ab150120) as secondary antibody. All antibodies were diluted with 3% BSA and 0.5% Tween in PBS. Around two hundred cells were observed and scored with a confocal laser scanning microscope (TCS-SP8, Leica) or a fluorescence microscope (BZ-9000, KEYENCE).

### Bisulfite sequencing analysis

To examine CpG methylation levels in the EF1α promoter region transduced by the pSicoR lentiviral vector, 500 ng of extracted genomic DNA was converted using the MethylEasy Xceed kit (Human Genetic Signatures). First-round PCR was performed by Quick Taq HS DyeMix (ToYoBo) using 25 ng of converted DNA in a 10 µl reaction mixture, then 0.5 µl of the first-round PCR product was used as template for nested PCR in a 25 μl reaction mixture. All primer sets and conditions are listed in Supplemental Table 6.

### Datasets

To examine the association between A3B expression and clinical outcomes, we used the Arkansas dataset (GSE4581)^7^, which consists of MAS5 normalized gene expression profiles generated using the Affymetrix U133Plus2.0 microarray platform and clinical information from the Multiple Myeloma Genomics Portal (MMGP) (http://portals.broadinstitute.org/mmgp/home).

### Statistical analysis

Descriptive statistics included absolute and relative frequencies for categorical data and median, mean, and range for numerical measurements. Mann-Whitney U test, Kruskal-Wallis test or Jonckheere-Terpstra trend test were used to evaluate the differences in continuous variables between two groups or more than three groups. Probabilities of OS were calculated based on Kaplan-Meier product limit estimates, and the OS outcomes of the two groups divided according to APOBECs expression levels were compared using the Logrank test. *P* values less than 0.05 were considered statistically significant. Multivariate analysis was performed using a Cox proportional hazard model for OS. Covariates with a *P* value < 0.2 factor in the univariate analysis were entered into the model. All statistical analyses were performed with EZR (Saitama Medical Center, Jichi Medical University), which is a graphical user interface for R (The R Foundation for Statistical Computing, version 3.0.2)^66^.

## Supplementary information

Supplemental Information

Supplemental Tables
