# Supplementary material for: Endogenous APOBEC3B Overexpression Constitutively Generates DNA Substitutions and Deletions in Myeloma Cells
Source: Sci Rep. 2019 May 9;9:7122. doi: 10.1038/s41598-019-43575-y (PMC6509214; doi:10.1038/s41598-019-43575-y)
Supplement: Supplementary file 1 — Supplemental Information [file 41598_2019_43575_MOESM1_ESM.pdf]

Supplementary Information for

# Endogenous APOBEC3B Overexpression Constitutively Generates DNA Substitutions and Deletions in Myeloma Cells

Hiroyuki Yamazaki[1], Kotaro Shirakawa[1], Tadahiko Matsumoto[1], Shigeki Hirabayashi[1, 2], Yasuhiro Murakawa[2, 3], Masayuki Kobayashi[1], Anamaria Daniela Sarca[1], Yasuhiro Kazuma[1], Hiroyuki Matsui[1], Wataru Maruyama[1], Hirofumi Fukuda[1], Ryutaro Shirakawa[4], Keisuke Shindo[1], Masaki Ri[5], Shinsuke Iida[5] and Akifumi Takaori-Kondo[1*]

From the [1]Department of Hematology and Oncology, Graduate School of Medicine, Kyoto University, Kyoto 606-8507, Japan, [2]RIKEN-HMC Clinical Omics Unit, RIKEN Baton Zone Program, Kanagawa, 230-0045, Japan, [3]RIKEN Preventive Medicine and Diagnosis Innovation Program, Kanagawa, 230-0045, Japan, [4]Department of Molecular and Cellular Biology, Institute of Development, Aging and Cancer, Tohoku University, Sendai 980-8575, Japan, [5]Department of Hematology and Oncology, Nagoya City University Graduate School of Medical Sciences, Nagoya, Japan.

# Figure S1

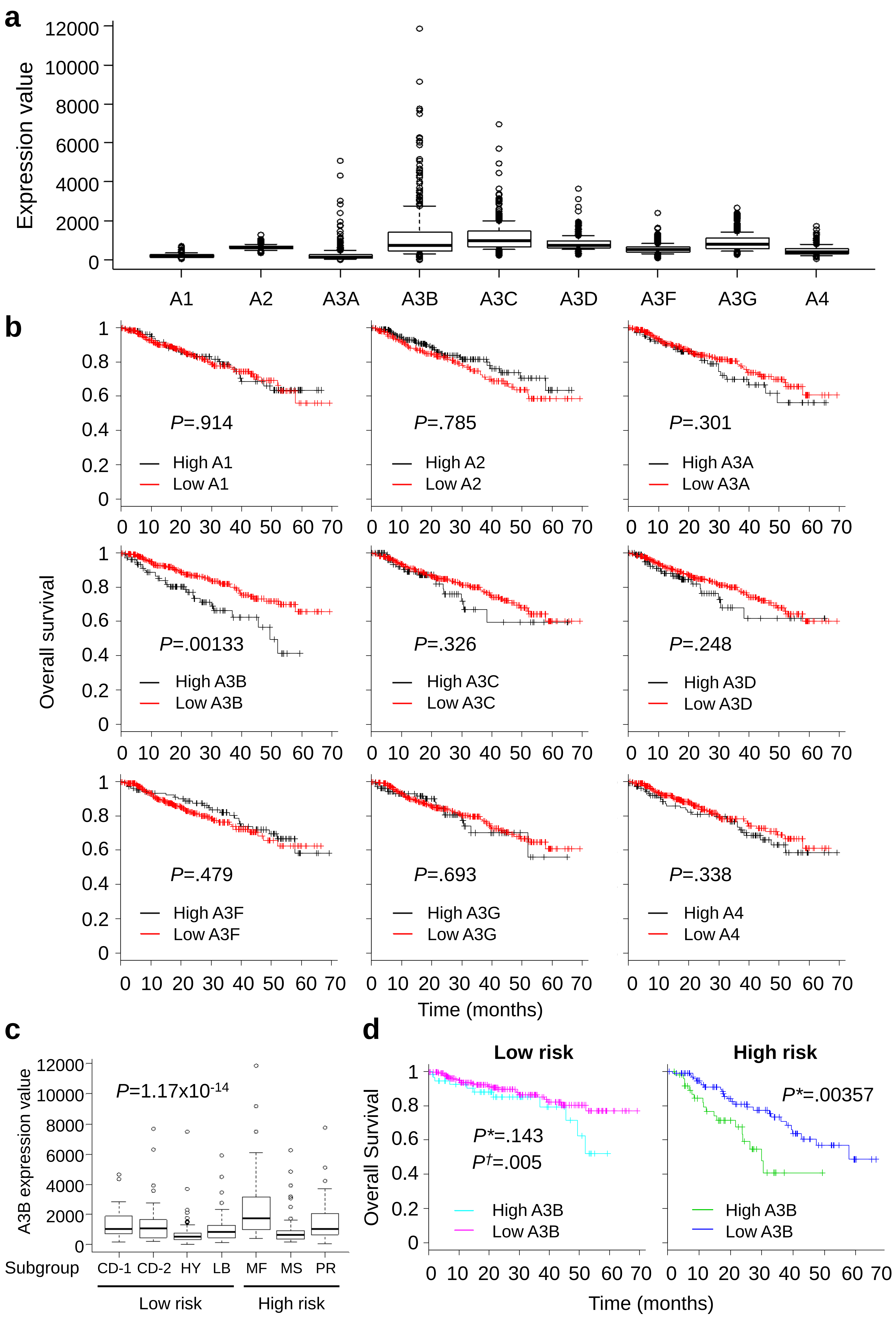


**Supplemental Figure 1.** Statistical analysis of APOBEC expression levels in myeloma patients. (**a**) Boxplot of APOBEC expression levels from a microarray dataset of 414 myeloma patients[7]. The normalized expression values of APOBEC1 (A1), APOBEC2 (A2), APOBEC3A-G (A3A-A3G) and APOBEC4 (A4) are shown with representations of the medians and the 10th and 90th percentiles. (**b**) Kaplan-Meier survival curves of the Arkansas cohort[7]. The patients were divided into two groups using the upper quartile of each APOBEC expression level as the threshold. The black and red graphs represent the high APOBEC and low APOBEC groups, respectively. The *P* values were calculated using the logrank test. (**c**) Boxplot of A3B expression levels in each subgroup (CD-1, CD-2, HY, LB, MS, MF and PR). The normalized expression values with representations of the medians and 10th and 90th percentiles are shown. The *P* values were calculated using the Kruskal-Wallis test. (**d**) Kaplan-Meier survival curves of each risk group. The low-risk group (left panel; HY, CD-1, CD-2, and LB; n=261 patients) and the high-risk group (right panel; MS, MF, and PR; n=153 patients) were each divided into high-A3B and low-A3B groups. The *P* values were calculated using the logrank test (*) and 45-month landmark analysis (†).

# Figure S2

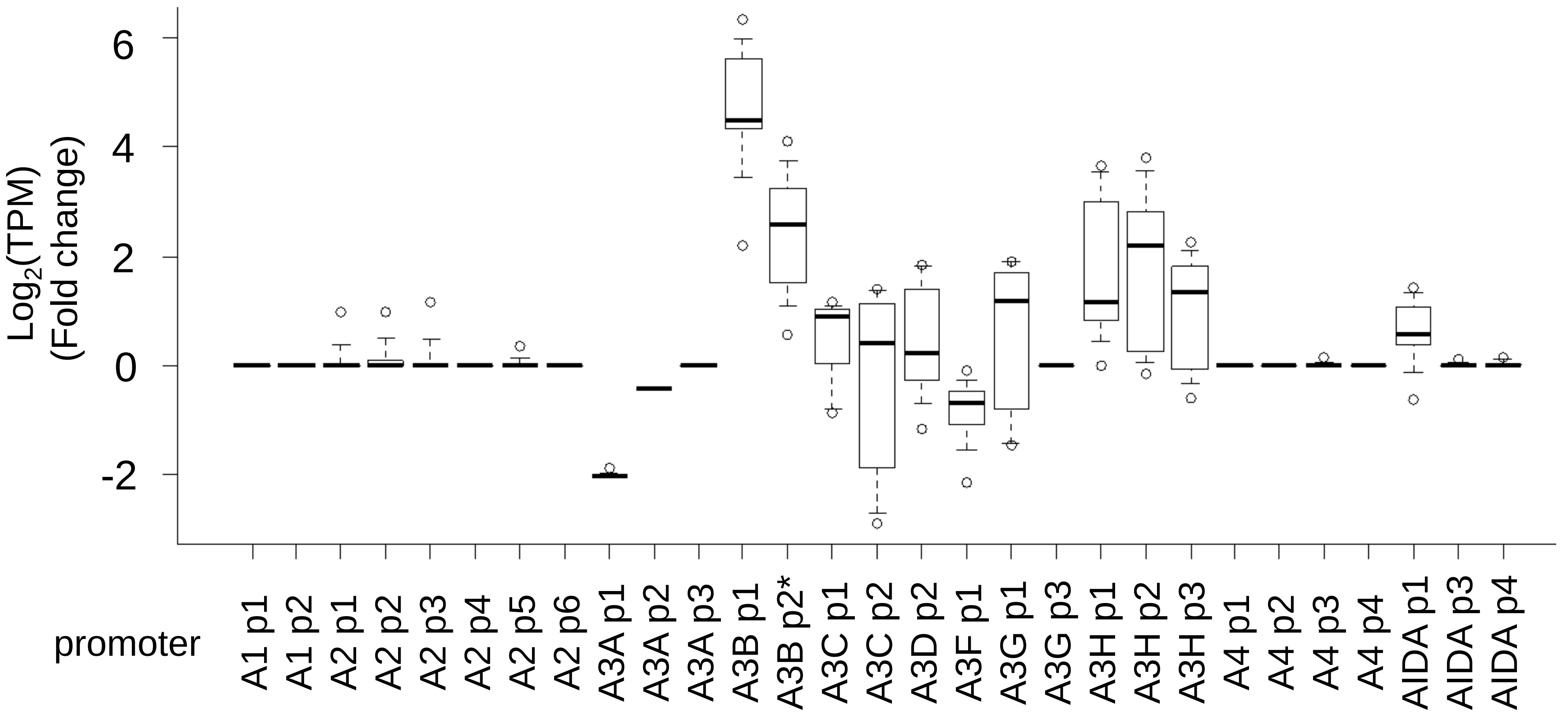


**Supplemental Figure 2.** Transcriptome of APOBEC family genes as determined by CAGE. Each TPM (tags per million) level of APOBECs in the seven myeloma cell lines is expressed relative to that in control CD19-positive cells.

# Figure S3

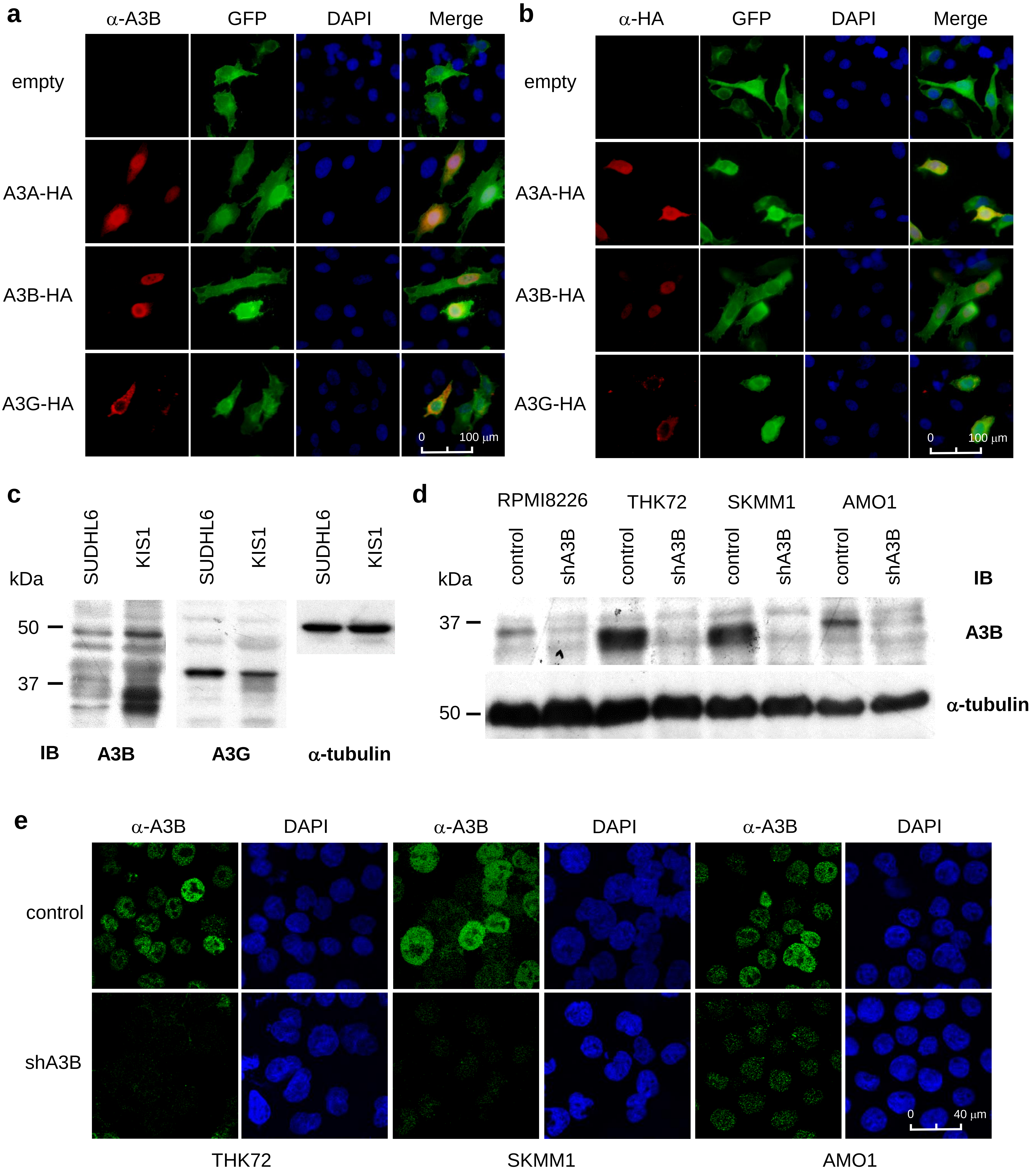


**Supplemental Figure 3.** Characterization of the anti-A3B antibody. (**a**, **b**) Fluorescence immunostaining of overexpressed HA-tagged A3A, A3B and A3G in HeLa cells using the anti-A3B antibody (a) and an anti-HA antibody (b). The cytoplasm was visualized by co-transfection with the S15-GFP plasmid. The images were obtained by fluorescence microscopy (magnification, 200×). (**c**) Immunoblot analysis of endogenous A3B and A3G in B-cell lymphoma cell lines. SUDHL6 cells were negative controls for A3B, whereas KIS1 cells were positive controls for A3B. α-Tubulin was evaluated as an internal control. (**d**) Immunoblot analysis of endogenous A3B in myeloma cell lines transduced with control or A3B shRNA lentivirus. α-Tubulin was evaluated as an internal control. The same populations of cells were analysed in Figures 2-5. (**e**) Fluorescence immunostaining with the anti-A3B antibody in THK72, SKMM1 and AMO1 cells transduced with either control or A3B shRNA lentivirus. The images were obtained by confocal fluorescence microscopy (magnification, 630×).

Figure S4

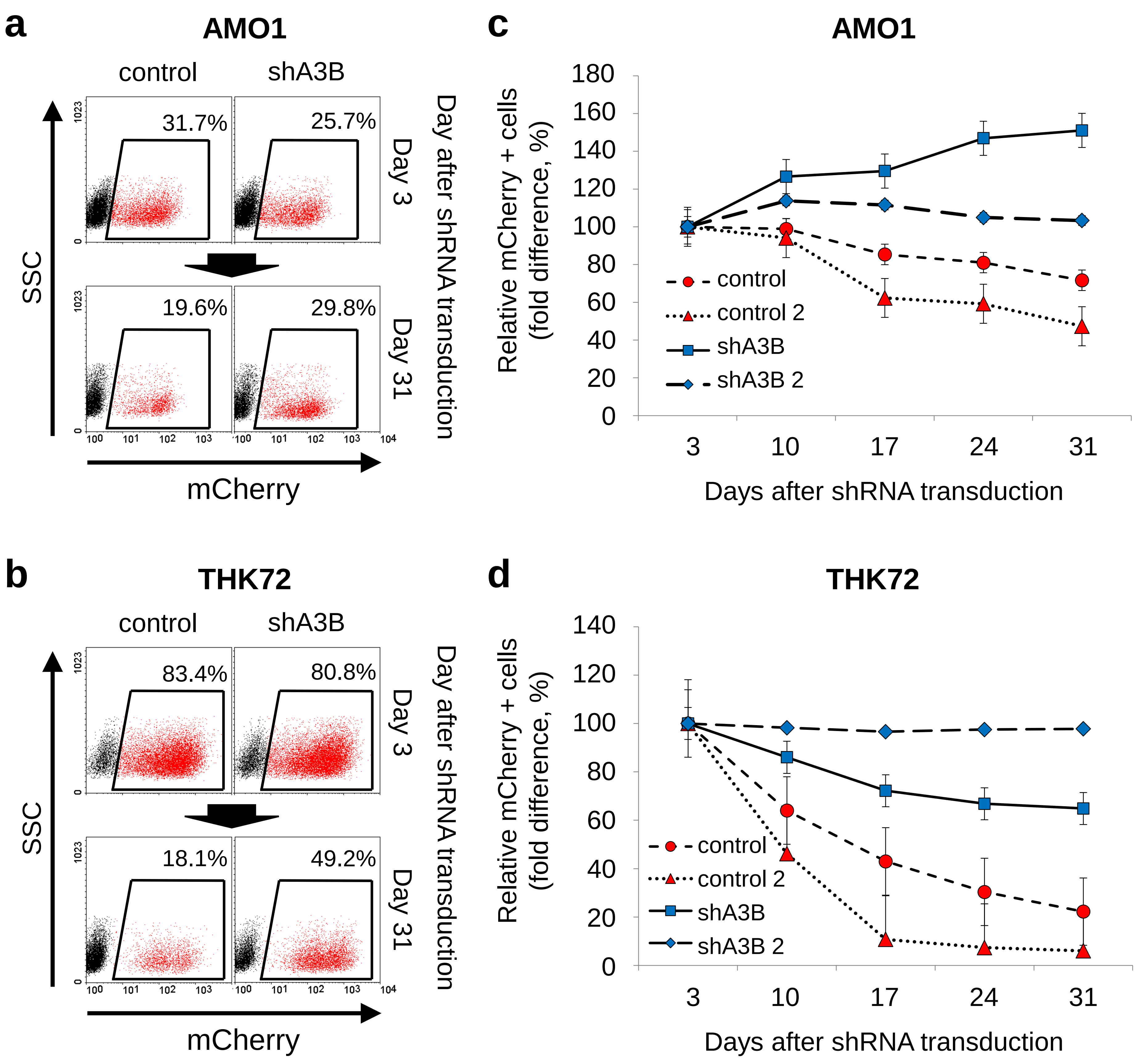


**Supplemental Figure 4.** APOBEC3B mediates the loss of function of exogenous genes in myeloma cells. (**a, b**) Flow cytometry of THK72 (a) and AMO1 (b) cells at 3 and 31 days after transduction with each mCherry-shRNA lentivirus. The numbers in boxes indicate the proportions of mCherry-positive cells among the live cells for each condition. (**c, d**) Time-dependent changes in the proportions of mCherry-positive THK72 (c) and AMO1 (d) cells among the live cells transduced with each shRNA virus as determined by flow cytometry.

# Figure S5

**a**

EF1α-mCherry-T2A-puroR Day 19

control (%)

| ↱ | A | C | G | T |
|---|---|---|---|---|
| A | | 666 (0.0386) | 392 (0.0227) | 106 (0.0061) |
| C | 983 (0.0569) | | 1000 (0.0579) | 626 (0.0363) |
| G | 34255 (1.9845) | 2218 (0.129) | | 1096 (0.0635) |
| T | 138 (0.0080) | 719 (0.0417) | 425 (0.0246) | |

total read bases, 1726170 bp
C>T|G>A mutation, 2.021%

shA3B (%)

| ↱ | A | C | G | T |
|---|---|---|---|---|
| A | | 565 (0.0327) | 525 (0.0304) | 144 (0.0083) |
| C | 716 (0.0415) | | 1203 (0.0697) | 897 (0.0520) |
| G | 1280 (0.0742) | 2238 (0.130) | | 848 (0.0491) |
| T | 129 (0.0075) | 542 (0.0314) | 429 (0.0249) | |

total read bases, 1725858 bp
C>T|G>A mutation, 0.1262%

**b**

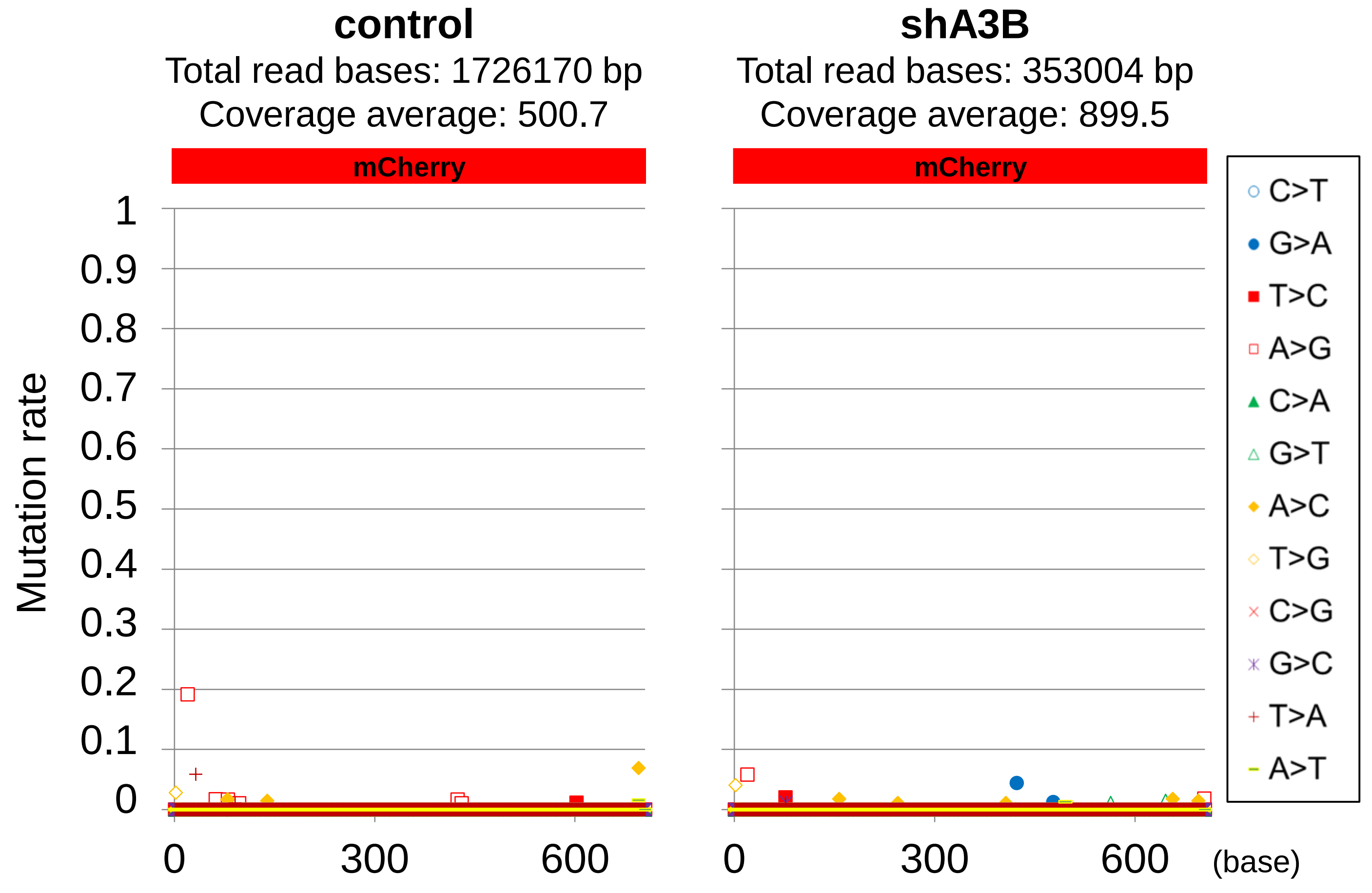


**Supplemental Figure 5.** Substitution frequency in 3D-PCR or conventional PCR products of lentivirally introduced genes in myeloma cells. (**a**) Mutation matrices of hyperedited EF1α-mCherry-T2A-puroR sequences. The sequence data were obtained by deep sequencing of the 3D-PCR products of mCherry-T2A-puroR genes derived from RPMI8226 genomic DNA at 21 days post transduction with each shRNA lentivirus. The first column indicates the bases before mutation, and the first line indicates the bases after mutation. The highlighted box indicates significant G-to-A substitutions. The sense strand of the EF1α-mCherry-T2A-puroR sequence was used as a reference. (**b**) Deep sequencing of conventional PCR products of mCherry genes derived from RPMI8226 genomic DNA at 21 days post transduction with each shRNA lentivirus. The Y axis indicates the proportion of each substitution in the total coverage, and the X axis indicates its location in the amplified mCherry gene.

# Figure S6

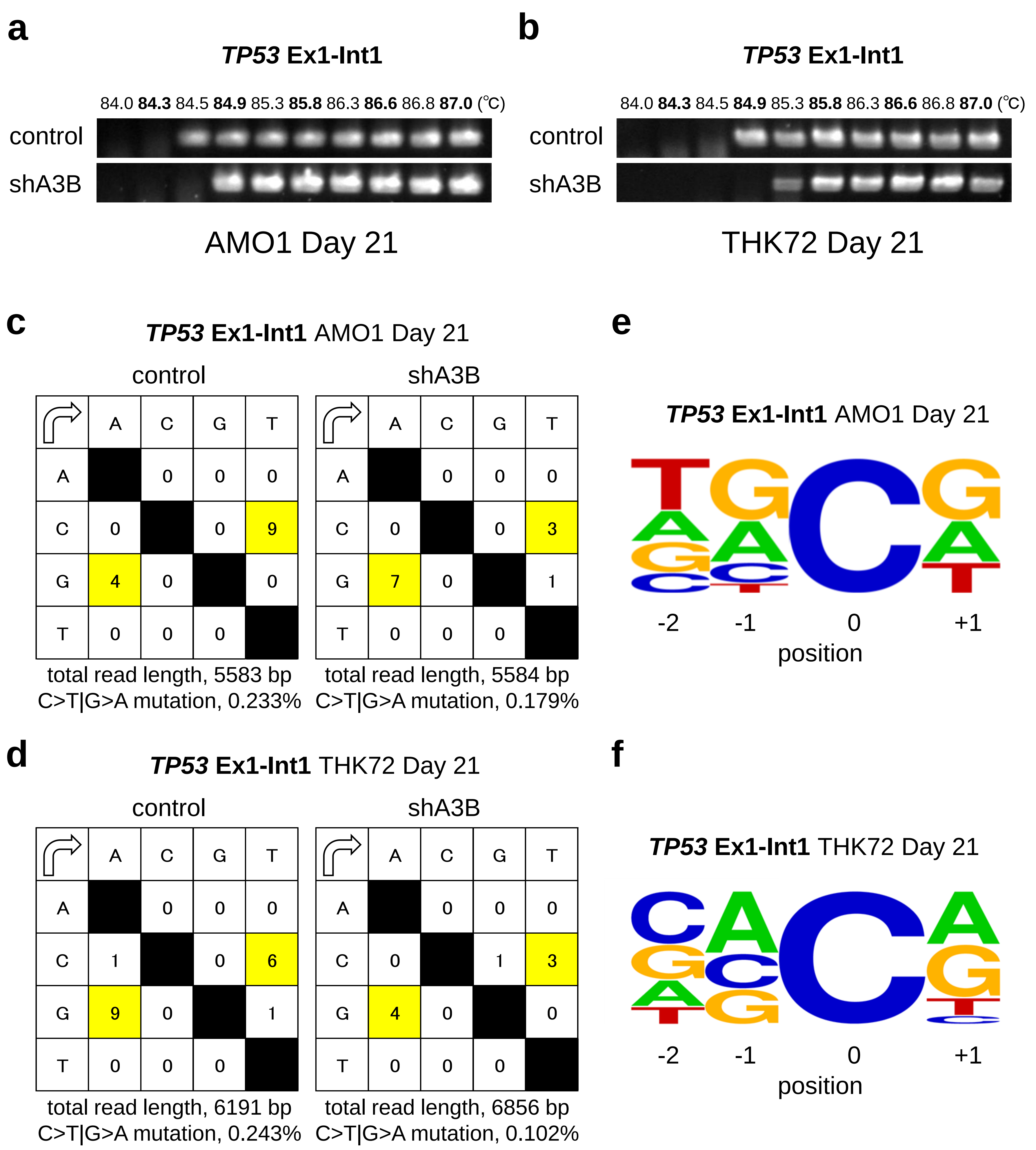


**Supplemental Figure 6.** Substitution frequency in 3D-PCR products of *TP53* in myeloma cells. (**a, b**) 3D-PCR of *TP53* from exon 1 (Ex1) to intron 1 (Int1) in genomic DNA obtained from AMO1 (a) and THK72 (b) cells at 21 days post transduction with control or shA3B lentivirus. (**c**, **d**) Mutation matrices of hyperedited *TP53* sequences from Ex1 to Int1 in genomic DNA obtained from AMO1 (c) and THK72 (d) cells at 21 days post transduction with each shRNA lentivirus. The sequence data were obtained by performing TA cloning and Sanger sequencing. The first column indicates the bases before mutation, and the first line indicates the bases after mutation. The sense strand of TP53 was used as a reference. C-to-T and G-to-A substitutions are highlighted. (**e**, **f**) Sequence logo created with WebLogo indicating the frequencies of nucleotides adjacent to C-to-T mutation sites in *TP53* Ex1-Int1 in AMO1 (e) and THK72 (f) cells.

**Supplemental Table 1.** MM/MGUS patient characteristics.
Abbreviations: NDMM, newly diagnosed multiple myeloma; RRMM, relapsed/refractory multiple myeloma; ISS, International Staging System; BJP, Bence Jones protein.

**Supplemental Table 2.** APOBEC3B genotypes of myeloma patients.
Data from healthy Japanese controls were obtained from a previous report[26]. The *P* values were calculated by Fisher's exact test.
Abbreviations: D/D, homozygous for the deletion allele; D/I, heterozygous; I/I, homozygous for the intact allele.

**Supplemental Table 3.** Univariate and multivariate analysis of OS segregated by patient APOBEC expression levels.
In the multivariate analysis, variables with a $P$ value < .2 in the univariate analyses were included in a Cox proportional hazard model. Only the variables remaining in the final model are shown.
Abbreviations: OS, overall survival; CI, confidence interval.

**Supplemental Table 4.** DNA sequences of mCherry genes derived from 3D-PCR products.
The boxes highlighted in yellow indicate point mutations, and those in pink indicate microhomology regions at the junction of the deletion.

**Supplemental Table 5.** Amino acid alignments of the mCherry protein translated from the cloned sequences.
The boxes highlighted in grey indicate amino acid substitutions or alterations. The shaded boxes indicate truncated sequences. $M^{71}$-$Y^{72}$-$G^{73}$ is the mCherry chromophore sequence.

**Supplemental Table 6.** List of primers and thermal cycling conditions for genotyping PCR, real-time PCR and 3D-PCR.
